# Nutritional status of children with cerebral palsy in Ghana

**DOI:** 10.4102/ajod.v13i0.1335

**Published:** 2024-07-31

**Authors:** Israt Jahan, Risad Sultana, Francis Laryea, Samuel Kofi Amponsah, Frederick Inkum Danquah, Mohammad Muhit, Sk. Md. Kamrul Bashar, Hayley Smithers-Sheedy, Sarah McIntyre, Nadia Badawi, Gulam Khandaker

**Affiliations:** 1 CSF Global, Dhaka, Bangladesh; 2 Asian Institute of Disability and Development (AIDD), University of South Asia, Dhaka, Bangladesh; 3 Central Queensland University, School of Health, Medical and Applied Sciences, Rockhampton, Queensland, Australia; 4 Korlebu Teaching Hospital, Accra, Ghana; 5 Health Information Department, Christian Health Association of Ghana, Accra, Ghana; 6 St. John of God College of Health, Duayaw Nkwanta, Ghana; 7 Cerebral Palsy Alliance Research Institute, Specialty of Child and Adolescent Health, Sydney Medical School, Faculty of Medicine and Health, The University of Sydney, Camperdown, New South Wales, Australia; 8 Grace Centre for Newborn Intensive Care, Sydney Children’s Hospital Network, Westmead, New South Wales, Australia; 9 Central Queensland Public Health Unit, Central Queensland Hospital and Health Service, Queensland, Australia; 10 Discipline of Child and Adolescent Health, Sydney Medical School, The University of Sydney, Camperdown, New South Wales, Australia

**Keywords:** malnutrition, stunting, underweight, cerebral palsy, disability, children, Ghana

## Abstract

**Background:**

Limited knowledge on nutritional epidemiology in Ghanaian children with Cerebral Palsy (CP) necessitates a comprehensive investigation for an improved understanding of malnutrition in this population.

**Objectives:**

We aimed to describe the epidemiology of malnutrition among children with CP in Ghana.

**Methods:**

The study used data collected as part of the Ghana CP Register (GCPR). The GCPR is an institution-based surveillance of children with CP aged < 18 years in Ghana. Between October 2018 and April 2020, *N* = 455 children with CP were registered. Data were collected on (i) weight, length or height, mid-upper-arm-circumference of children with CP; (ii) socio-demographic characteristics; (iii) motor type and topography, gross motor function classification system level (GMFCS); (iv) associated impairments; (v) educational and rehabilitation status for each child. Descriptive and bivariate analyses were performed.

**Results:**

Mean and standard deviation age of the registered children at assessment was 5.9 ± 4.1 years, and 42.1% were female. Two-thirds of the children had ≥ one form of undernutrition (underweight or severely underweight: 38.9%, stunted or severely stunted: 51.2%, thin or severely thin: 23.8%). In the adjusted analysis, low maternal education, GMFCS-IV, speech impairment and epilepsy significantly increased the odds of undernutrition among participating children (aOR: 2.6 [95% CI:1.3–5.4]; 2.2 [95% CI:1.0–4.8]; 2.0 [95% CI:1.1–3.6]; 2.9 [95% CI:1.1–7.5] respectively).

**Conclusions:**

The high malnutrition rate indicates an urgent need for nutrition interventions and translational research to improve nutritional status and prevent adverse outcomes among children with CP in Ghana.

**Contribution:**

Our study contributes important data and a framework to develop guidelines and evidence-based interventions for children with CP in Ghana.

## Introduction

Nutritional management is complex for children with cerebral palsy (CP) as the motor impairment caused by damage or lesion to the developing brain can adversely affect their food intake, digestion and metabolism (Aggarwal, Chadha & Pathak 2015), thus increasing their risk of malnutrition.

Cerebral palsy is the leading cause of childhood disability globally (Kakooza-Mwesige et al. 2017; Khandaker et al. 2019). Available data suggest that the prevalence of CP is two to three times higher among children in low- and middle-income countries (LMICs) such as Bangladesh and Uganda compared to high-income countries (HICs) such as Australia and Sweden (ACPR 2018; Kakooza-Mwesige et al. 2017; Khandaker et al. 2019). Children with CP in LMICs are susceptible to malnutrition as demonstrated by recent population-based data from Bangladesh, Indonesia, Uganda and Nigeria. More than half of the children with CP in those study cohorts were underweight and stunted (Jahan et al. 2019b, 2021a; Kakooza-Mwesige et al. 2015; Okunade 2018). A similar high prevalence was reported in one small-scale community-based intervention study in Ghana (Polack et al. 2018). The study also identified feeding difficulties and severe motor impairment as the predictors of undernutrition in that cohort (Polack et al. 2018).

Nutritional status undoubtedly affects the health and well-being of children with CP. Malnutrition was found to be significantly related to increased hospitalisation and decreased social participation in North American children (Samson-Fang et al. 2002). Furthermore, an overrepresentation of severe undernutrition was observed among children with CP who died from infectious causes in Bangladesh (Jahan et al. 2019a). Evidence-based nutrition interventions or support programmes could improve the conditions or prevent such adverse consequences of malnutrition (e.g. low survival probability, high risk of infection and hospitalisation, and low social participation).

Community-based nutrition interventions hold the potential to improve feeding skills, the nutritional status of children with CP and the quality of life of their caregivers as reported in Tanzania and Ghana (Donkor et al. 2019; Mlinda, Leyna & Massawe 2018). However, robust evidence regarding the epidemiology of malnutrition is crucial to appropriately identify children at the most risk and in need of nutrition intervention programmes for optimal resource use in LMICs. There is a knowledge gap in our understanding of the severity and potentially manageable predictors of malnutrition (e.g. socio-demographic factors, gross motor function limitations, feeding difficulties and access to rehabilitation services) among children with CP in LMICs from the West African region. In this study, we aimed to describe the epidemiology of malnutrition and identify the potential predictors of malnutrition among children with CP in Ghana.

## Research methods and design

This study was conducted as part of the Ghana CP Register (GCPR), an institution-based surveillance of children with CP.

### Participants and settings

The study participants were children with confirmed CP aged < 18 years registered into the GCPR between October 2018 and April 2020 from selected tertiary healthcare facilities located in the Eastern, Ahafo, Bono East and Ashanti regions. These centres are: (1) Salvation Army Rehabilitation Centre in Begoro, (2) St. John of God Hospital, Duayaw Nkwanta, (3) Holy Family Hospital in Techiman, Bono East and (4) Komfo Anokye Teaching Hospital in Kumasi, Ghana. These centres are the leading tertiary health care and rehabilitation facilities in the regions that cater to the service needs of patients from different parts of the country.

During routine healthcare services (at the above-mentioned facilities), if health service providers identified a child aged < 18 years with confirmed or suspected CP, then they referred that child to the GCPR team for detailed assessment following a structured protocol (Jahan et al. 2021b). A child was only registered in the CP Register (i.e. GCPR) if he met the case definition of CP used in the GCPR (Jahan et al. 2021b) and had a confirmed diagnosis. All children aged < 18 years with CP who visited the facilities (whether for their initial consultation or follow-up appointments) during the study period were eligible for registration in the GCPR, thus in this study.

### Data collection

Each child registered in the GCPR underwent detailed neurodevelopmental assessment by a multidisciplinary assessment team including a doctor, physiotherapist, occupational therapist and research officer. During this assessment process, the team collected data on selected variables using a standard protocol and case record form adapted from the global LMIC CP Register (GLM CPR) (Jahan et al. 2021b; Khandaker et al. 2015). Information was collected on (1) socio-demographic characteristics; (2) commonly known risk factors of CP; (3) motor type and topography using a standard protocol (Reid, Carlin & Reddihough 2011a, 2011b); (4) motor severity using the gross motor function classification level (GMFCS) (Palisano et al. 2000). The GMFCS has five levels and higher levels indicate more severe motor function impairment. For example, a child with GMFCS level I could walk independently in all settings whereas a child with GMFCS level V would need a wheelchair or assistive device and have limited stability of head and trunk postures or control over leg and arm movements (Palisano et al. 2000); (5) presence (yes or no) of associated impairments such as speech, hearing, intellectual, vision impairment and epilepsy; (6) anthropometric measurements and (7) education and rehabilitation status.

### Anthropometric data

At the time of registration in the GCPR, data on the following anthropometric measures were collected for each child: (1) weight in kilogram and (2) height or length in centimetres and (3) mid-upper arm circumference (MUAC) in centimetres for children aged < 5 years. Additionally, (4) the date of birth, (5) the date of assessment and (6) gender were documented. Three repeated measures (on the same day) were collected for all anthropometric measurements following the World Health Organization (WHO) guideline and the average was recorded (WHO 2008).

Weight was measured using a digital weighing scale. Tared weight (i.e. child’s weight = [weight of caregiver and child together] – [weight of caregiver only]) was measured for very young children and children who could not stand independently because of severe motor impairment. Length was measured for younger children (i.e. aged less than 24 months) and height was measured for children aged 24 months and older. Furthermore, knee height was measured for children who had joint contracture or scoliosis or involuntary movement (e.g. children with dyskinesia), and the full length or height was estimated using the following formula: height = (2.69 x knee height) + 24.2 (Stevenson 1995). Mid-upper arm circumference was measured using the standard MUAC tape.

### Interpretation of anthropometric data

All anthropometric data (i.e. weight, height and MUAC) were compared to the WHO reference population, and *z* scores were calculated according to the gender of the child using WHO Anthro and WHO AnthroPlus software. We used z scores for the following indices to assess the nutritional status of study participants: (1) weight-for-age (WAZ), (2) height-for-age (HAZ), (3) weight-for-height (WHZ), (4) BMI-for-age (BAZ) and (5) MUAC-for-age (MUACZ). The nutritional status of children was determined using the WHO standards (i.e. z score > +2SD = overnourished, −2SD to +2SD = normal, ≥ −3SD to < −2SD = undernourished and < −3SD = severely undernourished). Children with WAZ, HAZ, BAZ and WHZ or MUACZ < −2SD were therefore considered underweight, stunted (chronic undernutrition), thin and wasted (acute undernutrition), respectively, in the bivariate analysis.

### Statistical analysis

Kolmogorov–Smirnov test was used to determine the distribution of continuous data (e.g. age, income and all *z* scores). Furthermore, all continuous data were recoded into categories as needed. The age of the registered children was categorised into four groups, that is, 0–4, 5–9, 10–14 and 15–17 years. Descriptive statistics (e.g. mean with standard deviation (SD), median with interquartile range (IQR) and frequency with valid percentages) were reported to describe the cohort. Principal component analysis (PCA) of selected socio-demographic variables was completed to generate a composite score indicating the socioeconomic status (SES) of participating children. The variables included in the PCA were study participants’ household characteristics, number of household members, number of rooms used for sleeping in the house, household source of drinking water, type of toilet used, educational level of the mother, educational level of the father, employment status of the mother, employment status of the father and monthly family income. The composite scores from PCA were then recoded into three groups using the following cut-offs (e.g. < 25th percentile = low SES, 25th to < 75th percentile = middle SES and ≥ 75th percentile = high SES).

Inferential statistics were used to identify the potential risk factors of malnutrition among participating children. Cross tabulation with the Chi-square test or Fisher’s exact test was carried out to identify the significant differences in proportions. Parametric tests (e.g. *t*-test and ANOVA) were used to compare the mean WAZ and HAZ of children according to their socio-demographic and clinical characteristics. A *p*-value of < 0.05 was considered as significant. Unadjusted odds ratio (OR) and adjusted odds ratio (aOR) were calculated with 95% CI to measure the association and identify potential predictors of undernutrition among children with CP registered in the GCPR. Missing data for any variable was indicated using footnotes, and valid percentages were reported throughout the results. All data management and analyses were completed using SPSS version 26 (IBM Corporation, Chicago, IL).

### Ethical considerations

Ethical clearance to conduct the study was obtained from the Ethics Board of the Christian Health Association of Ghana (Reference no.: CHAG-IRB07022021) and the National Catholic Health Service, St. John of God Hospital (Reference no.: SJOGH/AFSR/19). The study was conducted according to the guidelines of the Declaration of Helsinki.

## Results

Between October 2018 and April 2020, 455 children with CP were registered from the study sites (mean (SD) age at recruitment 5.9 (4.1) years [95% CI: 5.5–6.3], 42.1% (*n* = 191) female).

### The overall nutritional status

Two-thirds of the children registered in the GCPR had at least one form of undernutrition. Among the participating children, 38.9% [95% CI: 34.0–43.8] were underweight (WAZ: ≥ −3SD to < −2SD) or severely underweight (WAZ: < −3SD), 51.2% [95% CI: 46.6–55.9] were stunted (HAZ: ≥ −3SD to < −2SD) or severely stunted (HAZ: < −3SD) and 23.8% [95% CI: 20.0–27.9] were thin (BAZ: ≥ −3SD to < −2SD) or severely thin (BAZ: < −3SD). Furthermore, among children aged < 5 years, 32.4% [95% CI: 26.6–39.1] and 41.2% [95% CI: 34.6–48.5] were wasted (≥ −3SD to < −2SD) or severely wasted (< −3SD) according to the WHZ and MUACZ, respectively. The mean (SD) and median [IQR] of the z scores have been summarised in Table 1.

**TABLE 1:** The overall nutritional status of children with cerebral palsy (*N* = 455).

Indicator	*N*	Mean	SD	Nutritional status, *n* (%)							
				Overnourished		Normal		Undernourished		Severely undernourished	
				*n*	%	*n*	%	*n*	%	*n*	%
WAZ	376†,‡	−1.5	2.2	15	4.0	215	57.2	60	16.0	86	22.9
HAZ	439	−2.2	2.8	20	4.6	194	44.2	77	17.5	148	33.7
BAZ	438	−0.4	3.0	73	16.7	261	59.6	38	8.7	66	15.1
WHZ	212‡,§	−0.9	3.2	36	16.9	108	50.7	21	9.9	48	22.5
MUACZ	191‡,§	−1.6	1.8	4	2.1	109	56.8	37	19.3	42	21.9

WAZ, weight-for-age z score; HAZ, height-for-age z score; BAZ, BMI-for-age z score; WHZ, weight-for-height z score; MUACZ, MUAC-for-age z score.

† , WAZ was calculated for children aged < 10.1 years (*n* = 379);

‡ , Missing data, *n* = 3 for WAZ, *n* = 16 for WHZ and *n* = 37 for MUACZ;

§ , WHZ and MUACZ were calculated for children aged < 61 months (*n* = 228).

### Factors related to undernutrition

#### Socio-demographic factors

Age: Undernutrition was significantly higher among children aged 10–14 years (84.9%) and 15–17 years (86.4%) when compared to younger children (*p* < 0.001). The WAZ and HAZ gradually deteriorated among children with an increase in their age. The mean (SD) of WAZ and HAZ among children aged 0–4 years versus 10–14 years was −1.3 (2.4) and −1.8 (3.0) versus −2.3 (1.5) and −3.5 (2.1) (*p* = 0.09 and *p* < 0.001, respectively).

Gender: The presence of at least one form of undernutrition was more common among male children (72.3%) than females (59.4%) (*p* = 0.004).

Maternal and paternal educational level: A significant overrepresentation of undernutrition was observed among children whose mothers and fathers never received any formal schooling or completed primary education than others in the cohort (*p* < 0.001 and *p* = 0.018, respectively). The mean (SD) of WAZ and HAZ was −1.0 (2.3) and −1.7 (2.9) for children whose mothers had completed secondary or higher education level, which decreased to −1.7 (2.1) and −2.9 (2.7) among children whose mothers had no formal schooling (*p* < 0.001 and *p* = 0.001, respectively). A similar relationship was observed between fathers’ educational level and child’s nutritional status.

Maternal and paternal occupation, family income and SES: The undernutrition rate was significantly higher among children whose mothers or fathers were unemployed at the time of the child’s birth (*p* = 0.014 and *p* = 0.002, respectively). Children from low and middle-SES families had significantly higher rates of undernutrition compared with children from high-SES families (*p* = 0.002) (Table 2 and Table 1-A1).

**TABLE 2:** Socio-demographic characteristics and nutritional status of participating children (*N* = 455).

Characteristics	*n*	%	Presence of at least one form of undernutrition†,‡					OR (unadjusted)
			Yes (*n* = 300)		No (*n* = 148)		*p* §	
			*n*	%	*n*	%		
**Age groups (years)¶**	-	-	-	-	-	-	< 0.001	-
0–4	225	50.1	150	67.6	72	32.4	-	Ref
9–May	149	33.2	84	57.1	63	42.9	-	0.6 [0.4, 1.0]
14–Oct	53	11.8	45	84.9	8	15.1	-	2.7 [1.2, 6.0]
15– < 18	22	4.9	19	86.4	3	13.6	-	3.0 [0.9, 10.6]
**Gender¶**	-		-		-		0.004	-
Female	191	42.1	111	59.4	76	40.6	-	0.6 [0.4, 0.9]
Male	263	57.9	188	72.3	72	27.7	-	Ref
**Mother’s educational level at the time of child’s birth¶**	-	-	-	-	-	-	< 0.001	-
No formal schooling	138	31.4	99	72.8	37	27.2	-	2.2 [1.3, 3.5]
Completed primary	136	30.9	105	77.2	31	22.8	-	2.7 [1.6, 4.6]
Completed secondary or higher	166	37.7	90	55.2	73	44.8	-	Ref
**Father’s educational level at the time of child’s birth¶**	-	-	-	-	-	-	0.018	-
No formal schooling	85	19.6	58	69.0	26	31.0	-	1.4 [0.8, 2.5]
Completed primary	134	30.9	100	75.2	33	24.8	-	2.0 [1.2, 3.2]
Completed secondary or higher	214	49.4	128	60.7	83	39.3	-	Ref
**Maternal employment status at the time of child’s birth¶**	-	-	-	-	-	-	0.014	-
Unemployed	62	13.7	50	80.6	12	19.4	-	2.3 [1.2, 4.4]
Employed	390	86.3	248	64.8	135	35.2	-	Ref
**Father’s employment status at the time of child’s birth¶**	-	-	-	-	-	-	0.002	-
Unemployed	15	3.4	15	100.0	0	0.0	-	Not Applicable
Employed	432	96.6	280	65.9	145	34.1	-	-
**Source of drinking water¶**	-	-	-	-	-	-	0.772	-
Unimproved††	36	8.1	25	69.4	11	30.6	-	1.1 [0.5, 2.3]
Improved††	408	91.9	269	67.1	132	32.9	-	Ref
**Sanitation¶**	-	-	-	-	-	-	0.185	-
Unimproved‡‡	125	27.7	78	62.4	47	37.6	-	0.7 [0.5, 1.1]
Improved‡‡	326	72.3	220	69.0	99	31.0	-	Ref
**SES¶**	-	-	-	-	-	-	0.002	-
Low	88	20.4	61	70.1	26	29.9	-	1.5 [0.9, 2.6]
Middle	121	28.1	95	79.2	25	20.8	-	2.5 [1.5, 4.1]
High	222	51.5	133	60.7	86	39.3	-	Ref

OR, odds ratio; SES, socio-economic status.

† , Row percentage (valid, excluding missing data);

‡ , Missing data (*n* = 7);

§ , Chi-square test (2-sided);

¶ , Missing data;

†† , Improved sources included piped water, tube well or borehole, protected dug well, protected spring water, rainwater, bottled water and sachet water. Whereas non-improved sources included unprotected dug wells, unprotected springs, tanker trucks or carts with small tanks and surface water;

‡‡ , Improved indicates toilet facilities with flush or pour flush to sewer system, flush or pour flush to septic tank system, flush or pour flush to pit latrine, ventilated improved pit (VIP) latrine and pit latrine with slab. Whereas, non-improved indicates toilet facility with flush or pour flush not to sewer or septic tank or pit latrine, pit latrine without slab or open pit, bucket, hanging toilet or hanging latrine and no facility or bush or field.

### Clinical factors

#### Predominant motor type and topography

Overall, the presence of at least one form of undernutrition was significantly higher among children with spastic CP (*p* = 0.035), especially, those who had quadriplegia. The mean (SD) of WAZ and HAZ was significantly lower among children with spastic quadriplegia compared to others in the cohort (*p* = 0.015 and *p* = 0.015, respectively) (Table 3, Table 2-A1).

**TABLE 3:** Clinical characteristics and nutritional status of participating children (*N* = 455).

Characteristics	*n*	%	Presence of at least one form of undernutrition, *n* (%)†,‡					OR (unadjusted)
			Yes (*n* = 300)		No (*n* = 148)		*p* §	
			*n*	%	*n*	%		
**Predominant motor type**								
Spastic	317	69.7	221	70.6	92	29.4	0.035	Ref
Dyskinesia	38	8.4	18	50.0	18	50.0	-	0.4 [0.2, 0.8]
Ataxia	52	11.4	30	57.7	22	42.3	-	0.6 [0.3, 1.0]
Hypotonia	48	10.5	31	66.0	16	34.0	-	0.8 [0.4, 1.5]
**Topography**								
Mono or hemiplegia	60	18.9	36	61.0	23	39.0	0.004	Ref
Diplegia	80	25.2	50	64.9	27	35.1	-	1.2 [0.6, 2.4]
Triplegia	36	11.4	21	58.3	15	41.7	-	0.9 [0.4, 2.1]
Quadriplegia	141	44.5	114	80.9	27	19.1	-	2.7 [1.4, 5.3]
**GMFCS level** ¶								
I	59	13.1	32	56.1	25	43.9	< 0.001	Ref
II	119	26.4	64	54.2	54	45.8	-	0.9 [0.5, 1.7]
III	44	9.8	27	61.4	17	38.6	-	1.2 [0.6, 2.8]
IV	154	34.2	116	76.8	35	23.2	-	2.6 [1.3, 4.9]
V	74	16.4	57	78.1	16	21.9	-	2.8 [1.3, 6.0]
**Timing of the brain injury**								
Pre- or peri-natal	363	79.8	235	66.0	121	34.0	0.399	Ref
Postnatal	92	20.2	65	70.7	27	29.3	-	1.2 [0.7, 2.0]
**CP diagnosis age (years)** ¶								
< 1	167	36.9	106	65.4	56	34.6	0.065	Ref
1–1.9	123	27.2	73	59.8	49	40.2	-	0.8 [0.5, 1.3]
2–4.9	125	27.7	93	75.0	31	25.0	-	1.6 [0.9, 2.7]
≥ 5	37	8.2	27	73.0	10	27.0	-	1.4 [0.6, 3.2]
**Presence of associated impairments**								
**Visual** ¶								
No	426	93.8	277	66.1	142	33.9	0.175	Ref
Yes	28	6.2	22	78.6	6	21.4	-	1.9 [0.7, 4.7]
**Hearing** ¶								
No	353	79.9	223	64.3	124	35.7	0.005	Ref
Yes	89	20.1	71	79.8	18	20.2	-	2.2 [1.2, 3.8]
**Speech** ¶								
No	125	29.5	61	50.8	59	49.2	< 0.001	Ref
Yes	299	70.5	221	74.2	77	25.8	-	2.8 [1.8, 4.3]
**Epilepsy** ¶								
No	383	87.6	242	64.2	135	35.8	0.001	Ref
Yes	54	12.4	47	87.0	7	13.0		3.7 [1.6, 8.5]
**Intellectual††**								
No	147	59.5	70	49.3	72	50.7	< 0.001	Ref
Yes	100	40.5	79	79.0	21	21.0	-	3.9 [2.2, 6.9]
**Number of associated impairments**								
None	135	29.7	64	49.6	65	50.4	< 0.001	Ref
One	173	38.0	121	70.3	51	29.7	-	2.4 [1.5, 3.9]
Two or more	147	32.3	115	78.2	32	21.8	-	3.6 [2.2, 6.2]

OR, odds ratio; GMFCS, gross motor function classification system level; CP, cerebral palsy.

† , Row percentage;

‡ , Missing data (*n* = 7);

§ , Chi-square test (2-sided);

¶ , Missing data;

†† , intellectual impairment could be assessed for *n* = 247 children.

#### Gross motor function classification system level

The presence of at least one form of undernutrition was lowest among children with GMFCS level II (54.2%) and highest among children with GMFCS level V (78.1%) (*p* < 0.001).

#### Timing of the brain injury and diagnosis age of cerebral palsy

No significant relationship was observed between the timing of the brain injury that probably caused CP, the age of CP diagnosis and the nutritional status of the children registered in the GCPR (*p* = 0.399 and *p* = 0.065, respectively).

#### Associated impairments

We observed a significant overrepresentation of undernutrition among children who had at least one impairment (70.3% – 78.2%) than those without any impairment (49.6%) (*p* < 0.001). When investigating specific types of impairments, the presence of undernutrition was significantly higher among those who had hearing impairment (*p* = 0.005), speech impairment (*p* < 0.001), intellectual impairment (*p* < 0.001) and epilepsy (*p* = 0.001). Both the WAZ and HAZ indicated similar findings (Table 3, Table 2-A1).

#### Predictors of undernutrition among children with cerebral palsy using adjusted analysis

Based on findings from the bivariate analysis, we included variables in the adjusted model (i.e. logistic regression) to identify potential predictors of undernutrition among children with CP. When adjusted for age, gender and SES, we found that maternal educational level, GMFCS level, presence of speech impairment and epilepsy were significant predictors of undernutrition among participating children in the GCPR. Children whose mothers had completed only primary education had 2.6 times (95% CI: 1.3–5.4) higher odds of having undernutrition compared to those whose mothers had completed at least secondary education level. Moreover, having a GMFCS level IV, the presence of speech impairment and epilepsy increased the odds of undernutrition among children by 2.2 (95% CI: 1.0–4.8), 2.0 (95% CI: 1.1–3.6) and 2.9 (95% CI: 1.1–7.5) times, respectively, among children when adjusted for other covariates (Table 4).

**TABLE 4:** Predictors of undernutrition among participating children (adjusted analysis).

Factors†	aOR [95% CI]	*p*
**Age group**		
0–4	Ref	
5–9	0.6 [0.4, 1.1]	0.116
10–14	1.8 [0.7, 4.4]	0.186
15–17	2.0 [0.5, 7.8]	0.316
**Gender**		
Female	Ref	
Male	1.6 [1.0, 2.6]	0.052
**Maternal education level**		
No formal schooling	1.8 [0.9, 3.7]	0.096
Completed primary	2.6 [1.3, 5.4]	0.009
Completed secondary or higher	Ref	
**SES**		
Low	0.9 [0.4, 1.8]	0.724
Middle	1.2 [0.6, 2.6]	0.590
High	Ref	
**GMFCS level**		
I	Ref	
II	1.3 [0.6, 2.7]	0.559
III	1.4 [0.5, 3.8]	0.451
IV	2.2 [1.0, 4.8]	0.044
V	2.5 [1.0, 6.4]	0.053
**Speech impairment**		
No	Ref	
Yes	2.0 [1.1, 3.6]	0.019
**Epilepsy**		
No	Ref	
Yes	2.9 [1.1, 7.5]	0.027

aOR, adjusted odds ratio; CI, confidence interval; SES, socio-economic status; GMFCS, gross motor function classification system level.

† , Factors found significantly associated with undernutrition in unadjusted bivariate analysis were included in the adjusted model following the forward selection method.

#### Presence of multiple forms of undernutrition

**FIGURE 1:**
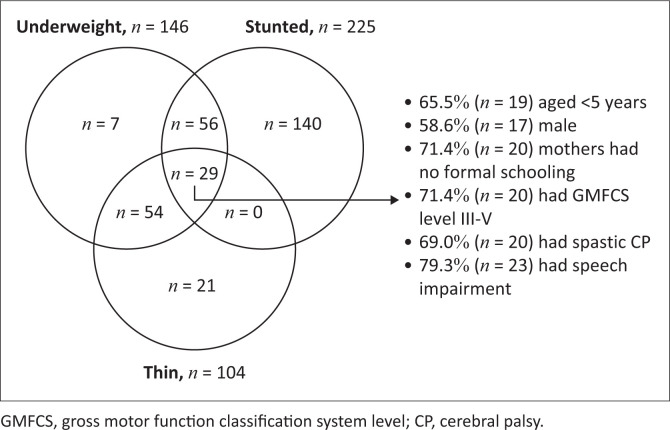
The presence of multiple forms of undernutrition among participating children.

The overlaps of different forms of undernutrition among children with CP have been illustrated in Figure 1. In our cohort, *n* = 29 children aged < 10.1 years had all three forms of undernutrition (i.e. underweight, stunted and thinness). When investigated further, we observed most of them had spastic CP (69.0%), tri or quadriplegia (60.0%), GMFCS level III-V (71.4%), speech impairment (79.3%) and intellectual impairment (50.0%).

## Discussion

The proportion of malnutrition among children with CP attending the healthcare facilities included in this study was alarmingly high. Two out of three children recruited in our study had at least one form of undernutrition. When compared with the national average, the proportion of underweight, stunting and wasting among under-five children with CP was substantially higher than the general population of the same age (40.6% vs. 11%; 46.4% vs. 19% and 32.5% vs. 5.0%, respectively) (Ghana Statistical Service [GSS], Ghana Health Service [GHS] 2015). Furthermore, the presence of multiple forms of undernutrition was commonly observed in our cohort. While the data presented here were collected from tertiary health care facilities and are not representative of the whole population, the burden of malnutrition observed in this group is still high when compared to other institution-based studies in different LMICs such as Uganda, Vietnam and Argentina (Kakooza-Mwesige et al. 2015; Karim et al. 2019; Ruiz Brunner et al. 2020). The findings from this research clearly indicate the vulnerability of these children registered in the GCRP towards poor growth, diminished immune system and health, severe motor function impairment and low survival probability in the long run (Aggarwal et al. 2015; Donkervoort et al. 2007; Jahan et al. 2019a; Namaganda et al. 2020).

To date, there has been very little research describing the epidemiology of malnutrition among children with CP in Ghana (Da Silva et al. 2022). Ghana CP Register has enabled us to explore the potential factors that could be addressed in interventions. Furthermore, the data generated could be used to design nutrition-sensitive and nutrition-specific interventions with immediate and long-term management to improve the health, motor function, social participation and survival of children with CP in Ghana. Our findings therefore contribute important epidemiological data to the current knowledge and understanding of malnutrition and the development of cost-effective strategies for nutritional management of children with CP in Ghana.

We observed an increasing trend in undernutrition among older children in our cohort. These findings are consistent with several institution-based studies in LMICs (Almuneef et al. 2019; Kakooza-Mwesige et al. 2015; Karim et al. 2019). In LMICs such as Ghana, children with CP often lack access to any early intervention and rehabilitation services (Al Imam et al. 2021), which also increases their risk for severe impairment of motor function as they grow older. This consequently increases their risk for associated feeding difficulties and digestive complications interfering with their dietary intake in general (Benfer et al. 2017; Polack et al. 2018).

Interestingly our data suggest a higher rate of undernutrition among male children with CP than females in the cohort. We also observed a positive relationship between maternal educational level, the SES of the families and the nutritional status of children registered in the GCPR. A similar pattern was reported in the latest demographic and health survey in Ghana (GSS 2023). The nationwide survey found a comparatively higher rate of underweight and stunting among children from low or middle SES than high SES. Similarly, malnutrition was more common among children whose mothers did not receive any formal education or had completed primary education when compared to those who had received higher education in Ghana (GSS 2023).

Although we have not been able to establish the causal relationship here, it is evident that these children could have benefited from early initiation of a nutrition intervention (e.g. feeding skill improvement, modification of food composition and tube-feeding) and rehabilitation services. Access to early intervention and rehabilitation services could improve the functional outcome (e.g. gross and fine motor function) of children with CP, which directly and indirectly affects their food consumption and nutritional status (Herrera-Anaya et al. 2016; Karim et al. 2021). In a recent study, children with higher muscle tone were reported to have significantly lower levels of fat mass, fat-free mass and muscle mass compared to children with lower muscle tone (Więch et al. 2020). Through rehabilitation, the nutritional status of those children could be improved. Although we did not find any direct association between the age of CP diagnosis, the timing of brain injury and the nutritional status of children registered in the GCPR, delayed diagnosis and low rehabilitation service uptake are commonly observed in LMICs (Al Imam et al. 2021; Jahan et al. 2021b). This also reduces the opportunity for early intervention to improve functional and health outcomes (including nutrition).

When adjusted for other variables, the severity of motor impairment, presence of epilepsy and speech impairment significantly increased the odds of undernutrition among participating children. Severe gross motor functional limitation has previously been reported to significantly increase the risk of malnutrition among children with CP in several other institution-based studies in Tanzania, Vietnam and Argentina, and community-based studies in Bangladesh, Indonesia and Ghana (Almuneef et al. 2019; Herrera-Anaya et al. 2016; Huysentruyt et al. 2020; Jahan et al. 2019b, 2021a; Kakooza-Mwesige et al. 2015; Karim et al. 2019; Polack et al. 2018). As in several literature, children with severe functional motor limitations often present with severe feeding difficulties and oropharyngeal dysphagia, which directly affects their nutritional status (Benfer et al. 2017; Polack et al. 2018). Furthermore, the high burden of undernutrition among children with speech impairment can indicate issues with oro-motor function and cognition among children with CP, which likely increases their vulnerability to dysphagia.

Evidently, those children with severe forms of CP could have benefitted from direct nutritional support for instance via enteral feeding (Ferluga et al. 2014). However, such nutritional interventions require careful monitoring to prevent any adverse outcome such as infection, especially among non-ambulant children. Furthermore, overfeeding is also a risk if not managed properly. Our findings also suggest a higher proportion of overweight or obesity among children with GMFCS level III-V compared to children with GMFCS level I-II, although we did not collect information regarding feeding intervention, such as tube feeding, in this cohort.

Although we generated crucial evidence essential to understanding the epidemiology of malnutrition and nutritional management among children with CP, our study has several limitations. Firstly, the GCPR utilises an institution-based survey methodology, which imposes bias in participant recruitment and hence not generalisable to larger groups or population-based data. However, the recruitment sites do provide tertiary healthcare services across the country, providing an important snapshot of the status of children accessing these services. Secondly, we used cross-sectional data and could not add a control group, for example, children without CP to compare the different clinical characteristics (e.g. eating disorders) and establish a causal relationship between CP and undernutrition among children in Ghana. Thirdly, there was a large number of missing data for the ‘presence of intellectual impairment’ (as diagnosis could not be confirmed for *n* = 186 children). In addition, several other variables had a few missing data. Nevertheless, as mentioned in the Statistical analysis section, we reported valid percentages throughout the results sections and added footnotes to indicate missing in respective tables. Fourthly, as part of the GCPR, we did not collect information about the dietary intake pattern of participating children and thus could not relate the nutritional status of those children with CP to their current food consumption. Lastly, because of resource constraints (e.g. equipment, trained personnel), we could not include advanced tools to measure nutritional status (e.g. bioelectrical impedance and skin-fold thickness to measure the body composition) although those provide a more precise indication of the nutritional status of children with CP (Hardy et al. 2018).

## Conclusions

In conclusion, the rate of malnutrition is alarmingly high among children with CP attending tertiary health care facilities in Ghana. Urgent action should be taken to ensure that those children are brought under existing and target-based CP-specific nutrition intervention programmes to prevent any adverse outcomes. Evidence-based nutritional guidelines and intervention strategies or programmes could be adopted at the healthcare facilities to support families in taking care of their children with CP. Our data could serve as a baseline to develop such guidelines and for future population-based epidemiological studies in Ghana. Also, the GCPR cohort could provide a framework to pilot nutritional intervention programmes and conduct translational research to identify potential intervention strategies that are feasible and cost-effective in LMIC settings such as Ghana.

## Appendix

**TABLE 1-A1:** Mean (SD) score of WAZ, HAZ and BAZ of children according to their socio-demographic characteristics (*N* = 455).

Sociodemographic factors	Mean (SD) WAZ†	*p* ‡	Mean (SD) HAZ	*p* ‡	Mean (SD) BAZ	*p* †
	*n* = 376		*n* = 439		*n* = 438	
**Age in years**						
0–4	−1.30 (2.41)	0.09§	−1.77 (2.96)	< 0.001§	−0.53 (3.58)	0.04§
5–9	−1.76 (1.76)		−2.05 (1.96)		−0.44 (2.31)	
10–14	−2.34 (1.54)		−3.47 (2.13)		−0.03 (2.04)	
15–18	Not Applicable		−5.86 (3.02)		1.28 (1.98)	
**Gender**						
Male	−1.74 (2.14)	0.008	−2.32 (2.69)	0.43	−0.60 (3.18)	0.05
Female	−1.14 (2.18)		−2.11 (2.86)		−0.05 (2.69)	
**Mother’s educational level**						
No formal schooling	−1.73 (2.06)	< 0.001§	−2.86 (2.68)	0.001§	−0.32 (2.56)	0.16§
Completed primary	−2.03 (1.87)		−2.4 (2.5)		−0.73 (2.79)	
Completed secondary or higher	−0.96 (2.27)		−1.68 (2.93)		−0.07 (3.31)	
**Father’s educational level**						
No formal schooling	−1.5 (2.18)	0.007§	−2.32 (2.73)	0.009§	−0.39 (2.66)	0.45§
Completed primary	−1.99 (1.77)		−2.85 (2.52)		−0.47 (2.46)	
Completed secondary or higher	−1.16 (2.26)		−1.92 (2.82)		−0.09 (3.18)	
**Maternal employment status**						
Unemployed	−2.07 (1.83)	0.03	−2.83 (2.63)	0.06	−0.49 (2.73)	0.76
Employed	−1.38 (2.21)		−2.14 (2.78)		−0.36 (3.04)	
**Paternal employment status**						
Unemployed	−2.37 (1.33)	0.12	−2.39 (2.78)	0.83	−0.6 (3.34)	0.80
Employed	−1.46 (2.18)		−2.22 (2.75)		−0.39 (2.99)	
**Source of drinking water**						
Non-improved¶	−1.53 (2.75)	0.99	−2.01 (3.33)	0.59	−0.91 (2.83)	0.29
Improved¶	−1.52 (2.11)		−2.27 (2.71)		−0.36 (3.02)	
**Sanitation**						
Unimproved††	−1.27 (2.37)	0.18	−1.99 (2.48)	0.24	−0.42 (2.66)	0.95
Improved††	−1.61 (2.07)		−2.33 (2.85)		−0.40 (3.07)	
**SES**						
Low	−1.66 (2.39)	0.01§	−2.0 (3.18)	< 0.001§	−0.77 (2.83)	0.29§
Middle	−1.98 (1.83)		−3.11 (2.39)		−0.11 (2.66)	
High	−1.24 (2.14)		−1.92 (2.68)		−0.40 (3.08)	

SES, socio-economic status; WAZ, weight-for-age; HAZ, height-for-age; BAZ, BMI-for-age.

† , WAZ was calculated for children aged < 10.1 years;

‡ , Independent *t*-test (2-sided);

§ , ANOVA (2-sided);

¶ , Improved sources included piped water, tube well or borehole, protected dug well, protected spring water, rainwater, bottled water and sachet water. Whereas, non-improved sources included unprotected dug wells, unprotected springs, tanker trucks or carts with small tanks and surface water;

†† , Improved indicates toilet facilities with flush or pour flush to piped sewer system, flush or pour flush to septic tank system, flush or pour flush to pit latrine, ventilated improved pit (VIP) latrine and pit latrine with slab. Whereas, non-improved indicates toilet facility with flush or pour flush not to sewer or septic tank or pit latrine, pit latrine without slab or open pit, bucket, hanging toilet or hanging latrine and no facility or bush or field.

**TABLE 2-A1:** Mean (SD) score of WAZ, HAZ and BAZ of children according to their clinical characteristics (*N* = 455).

Clinical characteristics	Mean (SD) WAZ†	*p* ‡	Mean (SD) HAZ	*p* ‡	Mean (SD) BAZ	*p* ‡
	*n* = 376		*n* = 439		*n* = 438	
**Predominant motor type**						
Spastic	−1.55 (2.17)	0.79§	−2.54 (2.84)	0.001§	−0.14 (2.9)	0.08§
Dyskinesia	−1.19 (2.33)		−0.94 (2.65)		−0.97 (3.77)	
Ataxia	−1.34 (2.09)		−1.47 (2.11)		−1.14 (2.57)	
Hypotonia	−1.47 (2.16)		−2.05 (2.56)		−0.58 (3.26)	
**Topography**						
Mono or hemiplegia	−1.25 (2.67)	0.015§	−2.0 (2.66)	0.015§	−0.35 (2.92)	0.79§
Diplegia	−1.33 (1.66)		−2.12 (2.69)		−0.19 (2.93)	
Triplegia	−0.73 (1.96)		−2.10 (2.72)		0.26 (1.93)	
Quadriplegia	−2.0 (2.16)		−3.13 (2.94)		−0.14 (3.1)	
**Timing of the brain injury**						
Pre or perinatal	−1.45 (2.05)	0.46	−2.33 (2.66)	0.13	−0.12 (2.92)	< 0.001
Postnatal	−1.65 (2.6)		−1.84 (3.11)		−1.35 (3.09)	
**CP diagnosis age (years)**						
< 1	−1.31 (2.25)	0.43§	−2.14 (2.83)	0.15§	−0.12 (3.36)	0.57§
1– < 2	−1.6 (1.97)		−1.98 (2.58)		−0.6 (3.02)	
2– <5	−1.58 (2.31)		−2.42 (2.78)		−0.48 (2.68)	
≥ 5	−2.02 (2.03)		−3.09 (2.88)		−0.29 (2.18)	
**Associated impairments**						
**Visual**						
No	−1.46 (2.18)	0.38	−2.14 (2.75)	0.005	−0.4 (3.04)	0.29
Yes	−1.9 (2.04)		−3.66 (2.64)		0.20 (2.26)	
**Hearing**						
No	−1.37 (2.16)	0.02	−2.00 (2.80)	< 0.001	−0.42 (3.08)	0.46
Yes	−2.01 (2.08)		−3.18 (2.37)		−0.15 (2.78)	
**Speech**						
No	−0.78 (2.30)	< 0.001	−1.6 (3.1)	< 0.001	−0.24 (3.05)	0.94
Yes	−1.79 (1.99)		−2.72 (2.45)		−0.22 (2.69)	
**Intellectual**						
No	−0.80 (2.35)	0.001	−1.05 (2.79)	< 0.001	−0.47 (3.2)	0.39
Yes	−1.92 (1.86)		−3.07 (2.62)		−0.13 (2.81)	
**Epilepsy**						
No	−1.39 (2.17)	0.01	−2.22 (2.64)	0.03	−0.24 (2.75)	0.84
Yes	−2.15 (1.92)		−3.25 (2.83)		−0.16 (3.5)	

CP, cerebral palsy; WAZ, weight-for-age; HAZ, height-for-age; BAZ, BMI-for-age.

† , WAZ was calculated for children aged < 10.1 years;

‡ , Independent t-test (2-sided);

§ , ANOVA (2-sided).
